# Serum EPO and VEGF levels in patients with sleep-disordered breathing and acute myocardial infarction

**DOI:** 10.1007/s11325-013-0801-z

**Published:** 2013-01-23

**Authors:** Wojciech Kukwa, Renata Glowczynska, Krzysztof J. Filipiak, Andrzej Kukwa, Grzegorz Opolski, Anna Budaj-Fidecka, Marcin Grabowski, Adam Galazka, Antoni Krzeski, Monika Kuzminska, Anna M. Czarnecka

**Affiliations:** 1 Department of Otolaryngology, Czerniakowski Hospital, Medical University of Warsaw, 19/25 Stepinska Street, 00-739 Warsaw, Poland; 2 1st Department of Cardiology, Medical University of Warsaw, Central University Hospital, 1a Banacha Street, 02 097 Warsaw, Poland; 3 Laboratory of Molecular Oncology, Department of Oncology, Military Institute of Medicine, 128 Szaserow Street, 04-141 Warsaw 44, Poland; 4 Department of Otolaryngology and Head and Neck Disease, University of Varmia and Masuria School of Medicine, 14 Zolnierska Street, 10-561 Olsztyn, Poland

**Keywords:** Erythropoietin, Myocardial infarction, Polysomnography, Sleep-disordered breathing

## Abstract

**Background:**

A high level of endogenous erythropoietin (EPO) may be associated with a smaller infarct size determined by the release of necrosis markers. Sleep-disordered breathing (SDB) is a well-known risk factor for cardiovascular diseases. In contrast, protective effects of SDB have also been described. The potential role of increased levels of EPO and vascular endothelial growth factor (VEGF) is suggested in this process. The study aimed to explore the EPO and VEGF serum levels in SDB and non-SDB patients during the acute phase of myocardial infarction.

**Methods:**

Thirty-seven patients undergoing successful primary percutaneous coronary intervention in the acute myocardial infarction have been examined for the levels of EPO, VEGF, and troponin I (Tn). In the following, patients had an overnight polysomnography to determine breathing disturbances during sleep.

**Results:**

Both on admission day (day 1) and day 3 of hospitalization, EPO levels showed statistically significant differences in both SDB-positive and SDB-negative patient groups (*p* = 0.003 and *p* = 0.018, respectively). There was no statistically significant difference in VEGF levels. No correlation was found between the EPO and Tn levels.

**Conclusions:**

SDB patients tend to have higher levels of EPO during acute myocardial infarction. No statistically significant differences in VEGF levels were observed.

## Introduction

In response to ischemia, mammalian cells express a variety of proteins, among which erythropoietin (EPO) plays a crucial role. EPO has long been identified as a primary regulator of erythropoiesis in bone marrow. EPO has also been reported to be expressed in the liver, lung, spleen, uterus, heart, testes, and neuroglia [1]. Furthermore, over the last decade EPO has also been recognized to play a role in a broad variety of processes in cardiovascular pathophysiology. In particular, the strong interactions of EPO with the nitric oxide pathway, apoptosis, ischemia, cell proliferation, and platelet activation appear to be of great interest for clinicians.

Serum EPO levels in patients with myocardial infarction were also investigated. In humans with acute myocardial infarction who had successful reperfusion with primary or rescue percutaneous coronary intervention (PCI), clinical trials with intravenous administration of EPO analogues did not show to reduce infarct size nor did it improve left ventricular ejection fraction. Prolonged EPO therapy after myocardial infarction (MI) in a large animal model was shown to be safe and lead to an increase in viable myocardium, increased vascular density, and was shown to prevent further deterioration of left ventricular function [2].

Cell culture models as well as animal models were used to prove that EPO administration may protect cardiomyocytes against cell death induced by ischemia and cytotoxic agents. In details, the antiapoptotic effect of EPO has been described in rat cardiac myocytes [3] and myoblasts subjected to hypoxia [4]. It was EPO that stimulated vascular endothelial growth factor (VEGF) gene expression.

It is well known that EPO release is highly induced under hypoxic conditions. For both renal and extra-renal EPO production, oxygen tension in tissues, not the red blood cell (RBC) count, primarily determines de novo EPO synthesis. Nocturnal hypoxemia-reoxygenation cycles that are typical to sleep-disordered breathing (SDB) may lead to an ischemic preconditioning conferring profound protection from infarction. Apneic episodes during sleep may in consequence promote certain cytokines production and release to the blood. Among them, EPO and VEGF may be key players in pre-conditioning mechanisms enabling myocyte survival.

The purpose of the present study was to investigate whether sleep apnea patients with acute myocardial infarction treated with successful PCI had different serum EPO and VEGF levels when compared to non-SDB patients and whether it correlated with the size of the infarction determined by the release of troponin.

## Material and methods

### Patients

**Fig. 1:**
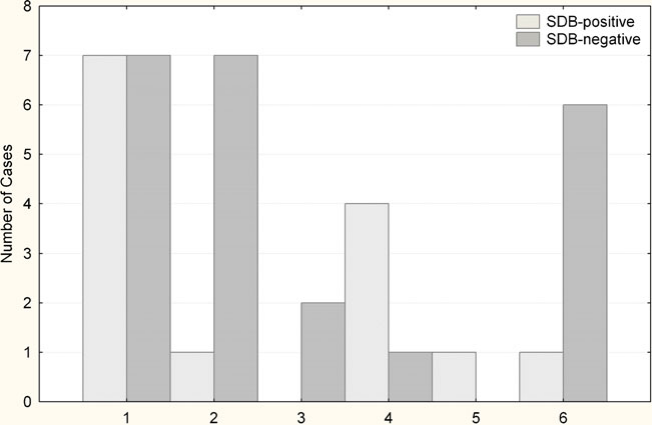
SDB and non-SDB patients according to the infarction location. *1* inferion, *2* anterior, *3* anterolateral, *4* inferolateral, *5* lateral, *6* inferoposterior

The study enrolled 37 men at ages below 65 years (mean age, 52.1 ± 6.5 years) admitted to the 1st Department of Cardiology at Medical University of Warsaw between February 2006 and December 2007. Acute myocardial infarction (AMI) was diagnosed according to the universal definition of myocardial infarction. The eligible patients were those men with first AMI who received successful primary PCI for the infarct-related coronary artery within 12 h from the onset of symptoms. All patients met inclusion criteria, none met exclusion criteria. Patients with previous myocardial infarction, previous coronary angioplasty, previous coronary artery bypass grafting, or heart failure were excluded to avoid the possible influence of proangiogenic growth factors released under these conditions. For each patient, the history of hypertension, diabetes, cigarette smoking, family history of premature coronary artery disease, or hypercholesterolemia was elicited. Studied groups were comparable in terms of epidemiological data (Table 1, Fig. 1).

**Table 1:** Cardiovascular risk relating to groups (mean; CI)

	SDB (*n* = 14) (38 %)	Non-SDB (*n* = 23) (62 %)	*p* value
	AHI ≥ 5	AHI <5	
Diabetes mellitus, *n* (%)	1 (7)	4 (17)	0.63
Arterial hypertension, *n* (%)	10 (71)	12 (52)	0.31
Dyslipidemia, *n* (%)	10 (71)	10 (43)	0.17
Smoker, *n* (%)	11 (78)	19 (82)	0.89
LDL, mg/dL	129.4 ± 23.5	128.6 ± 17.2	0.95
HDL, mg/dL	49.2 ± 6.9	46.1 ± 5	0.45
TG, mg/dL	172.3 ± 68.9	173.3 ± 39.6	0.97
RBC, mln/mm^3^	4.8 ± 0.2	5.6 ± 1.5	0.41
Hb, g/Dl	14.9 ± 0.5	15.1 ± 0.7	0.71
Age, years	53.3 ± 3.4	50.2 ± 2.4	0.83
Weight, kg	85.4 ± 9.6	84.1 ± 5.4	0.33
Height, cm	174.6 ± 4.7	175.5 ± 2.7	0.4
Hip, cm	102.4 ± 5.6	101.5 ± 3.4	0.73
Waist, cm	102.3 ± 7.6	98.3 ± 4.7	0.71
Neck, cm	41.6 ± 1.4	41.32 ± 1.4	0.24
BMI, kg/m^2^	27.9 ± 2.8	27.3 ± 1.9	0.62
AHI, events/h	18.7 ± 7.1	1.8 ± 0.6	<0.001
Minimum O_2_ saturation, %	83.9 ± 4	88.7 ± 1.9	0.04
SBP, mmHg	131.5 ± 18.4	129.4 ± 11.6	0.56
DBP, mmHg	80.2 ± 9.4	75 ± 5.9	0.42
HR, bpm	75.2 ± 7,6	79.7 ± 8	0.40
Killip Class	1.1 ± 0.2	1.2 ± 0.2	0.47
LVEF	49.5 ± 3.4	50.3 ± 2.9	0.77

*BMI* body mass index, *AHI* Apnea–Hypopnea Index, *LDL* low-density lipoprotein, *HDL* high-density lipoprotein, *TG* triglycerides, *RBC* red blood cells, *Hb* hemoglobin, *SB* systolic blood pressure, *DBP* diastolic blood pressure, *HR* heart rate, *LVEF* left ventricle ejection fraction

### Coronary angiography

All patients received a bolus of unfractionated heparin and dose of aspirin and clopidogrel before invasive procedure. Coronary angiography, as well as coronary angioplasty with stent implantation, was performed at admission in every patient. Coronary angiography and PCI were carried out via the femoral or radial approach. Standard angiography, with ≥4 views of the left coronary artery system and 2 views of the right coronary artery, was preformed and then used for interpretation.

### Laboratory examinations

Troponin I was assessed by a central laboratory using standardized methods. Blood samples for other laboratory parameters were collected on admission day and on day 3 of hospitalization. Those blood samples were obtained and transferred into sterile serum vials and immediately stored on ice. Within 30 min, samples were centrifuged at 1,000×*g* for 15 min and then stored in aliquots of 100 μL at −80 °C. The serum concentration of EPO and VEGF was measured by a commercially available and standardized ELISA kit with a specific monoclonal antibody (Quantikine High Sensitivity, R&D Systems). The assay was performed according to the manufacturer’s instructions. Two aliquots of each blood sample were assessed in duplicates simultaneously in each assay.

### Polysomnography

All-night complete polysomnography (Nicolet UltraSom ™) was performed at a reference center for sleep disorders at Department of Otolaryngology at Czerniakowski Hospital between 30 and 60 days after the coronary event and then reviewed by an experienced physician. The study included analysis of electrocardiography (ECG), electroencephalography, electromyography, and electrooculography. Thoracic and abdominal respiratory movements were recorded by respiratory inductance plethysmography and arterial oxyhemoglobin saturation by oximetry. Nasal and oral airflow were registered using a thermistor.

Apnea was defined as a cessation of airflow for at least 10 s, and hypopnea was defined as a 50 % or greater decrease in the amplitude in either of the two respiratory effort signals, resulting in a decrease of at least 4 % in arterial oxyhemoglobin saturation. The following polysomnographic parameters were assessed: Apnea–Hypopnea Index (AHI), mean/minimal oxygen saturation, minimal desaturation, mean apnea duration, total sleep duration, and arousal index. Sleep-disordered breathing was diagnosed when AHI was equal or higher than five episodes per hour.

### Ethics committee

The study protocol was reviewed and approved by the Ethics Committee of the Medical University of Warsaw (KB/233/2005), and written informed consent was obtained from each subject at enrollment.

### Statistical analysis

Patients were divided into two groups according to their AHI score obtained from the polysomnography. Group 1 was the SDB-positive group with AHI ≥ 5 (*n* = 14, AHI range 5.5–48.7), and group 2 was the SDB-negative group with AHI < 5 (*n* = 23, AHI range 0.0–4.1).

Statistical analysis was performed using the STATISTICA 8.0 software (StatSoft Inc.). The Kolmogorov–Smirnov test was used for assessing the normality of the sample distribution.

The continuous variables were tested for the statistically significant differences using Student’s *t* test, Mann–Whitney test or Fisher exact test when appropriate. The differences between the groups were presented on the box-and-whisker diagram indicating mean, 95 % confidence interval of the mean (CI), and range. Spearman rank correlation coefficients were computed to estimate possible interrelations.

A *p* value below 0.05 was considered statistically significant. All *p* values given are two-tailed.

## Results

**Fig. 2:**
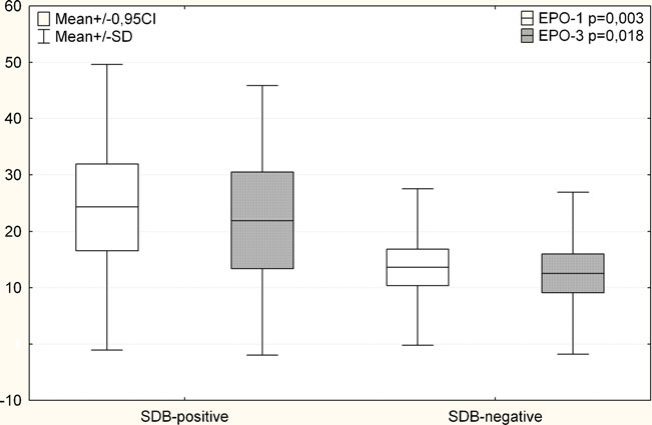
EPO levels in SDB and non-SDB patients on day 1 and day 3. (mean ± SD; 0.95 CI)

Both admission day and day 3 of hospitalization EPO levels were statistically significantly different in SDB-positive and SDB-negative patient groups (*p* = 0.003; *p* = 0.018; Fig. 2). The SDB-positive group had a higher mean of EPO levels on both days (day 1 mean, 24.3 ± 7.7; day 3 mean, 21.9 ± 4.0) compared to the EPO levels in the SDB negative group (day 1, 10.4 ± 3.2; day 3, 12.5 ± 3.4).

**Fig. 3:**
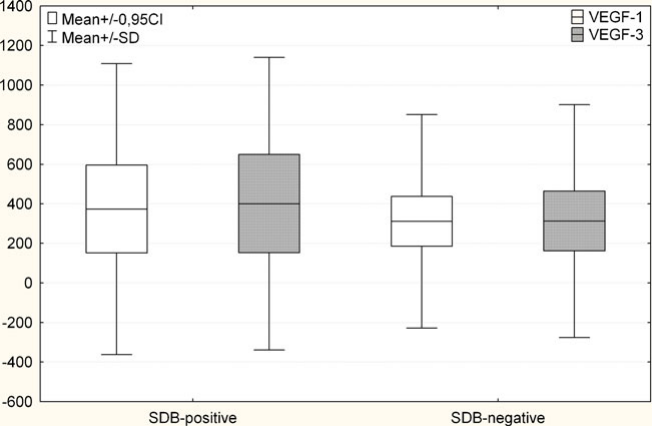
VEGF levels in SDB and non-SDB patients on day 1 and day 3 (mean ± SD; 0.95 CI)

The SDB-positive group had a higher mean of VEGF levels on both days (day 1 mean, 373.6 ± 222.4; day 3 mean 400.6 ± 248.8) compared to the VEGF levels in the SDB-negative group (day 1, 310.8 ± 126.3; day 3, 312.9 ± 151.3; Fig. 3). Mean calculated values were not statistically significantly different for both measurements (day 1, *p* = 0.98; day 3, *p* = 0.7).

The maximum Tn levels were also not statistically different in the patients’ subgroups (56.8 ± 42.8 vs 59.1 ± 27.6; *p* = 0.32). Moreover, the AHI showed no statistically significant correlations with EPO, VEGF, and Tn levels. Maximum Tn and EPO levels showed no correlation.

## Discussion

During the past two decades, sleep-disordered breathing (SDB) has been strongly associated with several cardiovascular disorders. Growing evidence supports the unfavorable influence of SDB on the cardiovascular system. In particular, sleep-disordered breathing may deteriorate the course of hypertension, coronary artery disease, congestive heart failure, stroke, pulmonary hypertension, and induce arrhythmias [5]. Obstructive sleep apnea increases the risk of coronary events or death from cardiovascular causes [6]. Cardiovascular disease patients with untreated severe SDB are also expected to have worse cardiovascular outcomes. Among patients with coronary artery disease and acute myocardial infarction, the prevalence of sleep apnea is as high as 65 % [7, 8].

In a whole body perspective, negative influence of SDB on the cardiovascular system is a result of hypoxia, sympathetic activation, swings in intrachoracic pressure during ineffective inspiratory efforts, oxidative stress, and activation of inflammatory cellular processes as well as increased glucose intolerance. On the contrary, protective effects of SDB have also been described. Lavie et al. hypothesized that nocturnal hypoxemia-reoxygenation cycles may lead to an ischemic preconditioning conferring profound protection from infarction [9]. Similarly, Koch et al. suggested that sleep apnea may be neuroprotective [10]. Hypothetically, the cardioprotection in patients with SDB might be a result of two different mechanisms. The first hypothesis states that as a result of repetitive hypoxia the coronary collateral circulation may be significantly developed. In particular, Steiner et al. using angiography found that apneic patients have developed multiple coronary collateral vessels [11]. The second hypothesis states that preconditioning of the myocardium by recurrent hypoxia may result in specific metabolic changes in the cardiomyocytes and result in increased hypoxia and acidosis tolerance. Apneic episodes during sleep may in consequence promote cytokines, chemokines, and growth factors’ overexpression and subsequent release into the blood. Among those, it is EPO and VEGF that are expected to be key players in pre-conditioning mechanisms responsible for myocyte survival [12].

The pathophysiological significance of VEGF activation in SDB is speculative. Some, but not all, investigations have reported a correlation between the severity of SDB (as measured by the apnea/hypopnea index) and VEGF levels. Nevertheless, in multiple reports patients with sleep apnea have increased serum and plasma levels of VEGF [13–15]. In this paper, the authors found no statistically significant difference in VEGF levels of SDB-positive and SDB-negative patients. The authors theorize that this may be due to the effect of a small sample size (see Fig. 3).

At the same time, conflicting reports on the EPO level in SDB patients were published. Goldman et al. found no relation between the degree of hypoxemia and serum EPO concentrations [16]. On the contrary, Winnicki et al. and Xu et al. demonstrated that EPO levels in sleep apnea patients are significantly higher than in normal adults, and that it may be decreased by successful SDB treatment [17, 18]. It is therefore hard to speculate on EPO release as a general phenomenon in SDB patients. Nevertheless, the findings of this study show that in AMI patients EPO levels on both days were statistically significantly higher in the SDB-positive group compared to the SDB-negative group. To the authors’ best knowledge, there are only three studies showing endogenous levels of EPO in MI patients.

Namiuchi et al. have shown that in MI patients who underwent successful primary PCI within 12 h from the onset of infarction symptoms, high serum EPO level was associated with a smaller infarct size as determined by the cumulative creatine kinase (CK) release. Furthermore, a stepwise multiple regression analysis revealed that the serum EPO level was an independent predictor for the cumulative CK release [19]. This was not confirmed by Ferrario et al., who observed no correlation between the EPO concentration and measured indexes of myocardial damage or necrosis in patients with AMI [20]. Niccoli et al. have described that angiographic and ECG no-reflow, in patients with first ST elevation myocardial infarction undergoing primary percutaneous coronary intervention, correlated with lower EPO levels at univariate analysis [21]. In the present study, there was no correlation between EPO and VEGF levels and the myocardium necrosis marker Tn.

At this moment, the authors stress that more questions on MI and OSA codependence remain to be answered in a large population study. Nevertheless, our research demonstrates that patients with sleep-disordered breathing tend to have higher EPO levels during acute myocardial infarction. Also, the levels of VEGF, that were never studied in such a group of patients [22], tend to be higher in SDB-positive patients, but the differences were not statistically significant. This may suggest a cardioprotective role of sleep apnea during AMI that takes place through hypoxic preconditioning.

In summary, the authors believe that this work would further support the hypothesis that SDB, apart from its negative influence on the cardiovascular system, might paradoxically trigger some protective mechanisms. Additional research on larger groups of patients is necessary to confirm this hypothesis

## Limitations of the study

1. There are several limitations of the study. First of all, the PSG recordings were carried out 1 to 2 months after the MI. Therefore, we cannot definitely determine the severity of SDB at the time of MI. Hypothetically, some patients could have developed the symptoms of congestive heart failure following the infarction, and those could have affected the diagnosis of SDB. To our best knowledge, there is only one study showing the fluctuation of SDB severity after acute coronary syndromes (ACS) [23]. According to Schiza et al., interestingly, the AHI level is stable during the first month after the ACS and then decreases over the next 5 months. It seems that PSG carried at 1–2 months after MI represents SDB that is not affected additionally by acute heart failure, and this would be desired by the study design. Moreover, in our study, patients had the left ventricular ejection fraction examined on the follow-up visit 3 months after the MI, and none of the patients had a significant decrease of the LVEF compared to the first measured value. This enables us to conclude that MI did not significantly influence the long-term NYHA status of the patients and SDB severity as a result. It should not be forgotten that an important limitation of the study by Schiza et al. is that the authors excluded patients with moderate and severe SDB as they focused on the sleep architecture.
2. The study is confined to the endogenous EPO levels, while clinical studies investigate the effect of EPO analogues (i.e., darbepoetin) on LVEF or infarct size [24]. The effect of exogenous EPO administration and endogenous EPO release cannot be directly compared as it has been proven that analogues differ according to receptor binding specificity and binding Km and, finally, doses administered in trials are not comparable with the physiological serum EPO levels [25].
3. Another limitation of our study was the serum VEGF level measurement method. Most authors agree that the plasma VEGF levels are more stable during measurements and, therefore, should be used [26], but both serum and plasma levels have been reported in studies that have been published up until today [27]. In this project, the serum level measurement was used, but we believe that it has not influenced the statistical analysis significantly as the relative levels were compared between groups, and the absolute values were not considered. Moreover, the clotting time is the most important factor influencing VEGF measurement in serum [28]. In our study protocol, this time interval was constant and no longer than 30 min. Therefore, it has not substantially influenced the patient-to-patient comparison.
4. Finally, the number of patients of this pilot study was too small to analyze the data in more detail and perform multiple regression analyses. No correlations with both the AHI or oxygen saturation levels could have been established, and this may be the result of the small number of patients enrolled in this pilot study. Therefore, this and other important questions remain to be answered in further, larger clinical studies.
